# Induction of Cytoplasmic dsDNA and cGAS-STING Immune Signaling After Exposure of Breast Cancer Cells to X-ray or High-Energetic Carbon Ions

**DOI:** 10.1016/j.adro.2025.101783

**Published:** 2025-04-07

**Authors:** Cristina Totis, Nicole B. Averbeck, Burkhard Jakob, Maik Schork, Gaia Volpi, Dennis F. Hintze, Marco Durante, Claudia Fournier, Alexander Helm

**Affiliations:** a GSI Helmholtzzentrum für Schwerionenforschung GmbH, Darmstadt, Germany; b Department of Biology, Technische Universität Darmstadt, Darmstadt, Germany; c Biology and Biotechnology Department, University of Pavia, Pavia, Italy; d Institute of Condensed Matter Physics, Technische Universität Darmstadt, Darmstadt, Germany; e Department of Physics “Ettore Pancini,” University Federico II, Naples, Italy

## Abstract

**Purpose:**

Radiation therapy can trigger activation of the cyclic GMP-AMP synthase (cGAS)- Stimulator of interferon genes (STING) axis via cytoplasmic dsDNA fragment induction. The activation of cGAS-STING initiates innate immune signaling mediated by interferon type I that can contribute to eradicate the malignancy. The effect was shown to depend on the fractionation scheme employed. We hypothesized that the innate immune response can also depend on radiation quality because densely ionizing radiation, such as carbon ions, have different effects on DNA lesion quality.

**Methods and Materials:**

We exposed an in vitro 4T1 breast cancer model to either photons or carbon ions and measured the clonogenic survival of cells with the colony-forming assay. The occurrence of cytosolic dsDNA fragments was assessed via immunofluorescence, whereas the expression and release of interferon-β by quantitative reverse transcription polymerase chain reaction and enzyme-linked immunosorbent assay. Bulk RNA sequencing was used to investigate global radiation-induced changes in gene expression.

**Results:**

We show here that carbon ions induced a significantly higher yield of cytosolic dsDNA fragments per unit dose as compared to photons. The higher efficiency also translated in expression and release of interferon-β by the tumor cells. The rate of cytoplasmic dsDNA foci as well as interferon-β release increased with doses up to 20 Gy and no differences for a fractionation scheme (3 × 8 Gy) were found as compared to the single high doses (20 or 24 Gy) of photons.

**Conclusions:**

In conclusion, we found that the release of interferon-β after radiation increases with the radiation dose up to 20 Gy and that carbon ions have the potential to elicit a strong innate immune signaling.

## Introduction

Triple-negative breast cancer remains a challenge for treatment because of its resistance to standard treatments, especially chemotherapy and radiation therapy.^1^ As for radiation therapy, the location of the tumor represents a further challenge as lung and heart are sensitive organs at risk and may receive a certain dose during treatment, increasing the risk for late adverse cardiovascular effects.^2^ Because of a higher precision and better sparing of healthy tissue, particle therapy using protons is considered an alternative and has been shown to be safe and feasible, reducing the dose to nontarget structures while optimizing target coverage, but long-term clinical data are yet scarce.^3^ Carbon ion radiation therapy (CIRT) is also attractive because it offers comparable precision as compared to protons plus an increased biological effectiveness as compared to photons.^4^ This increased biological effectiveness results in an improved control of radioresistant tumors and may be also more immunogenic.^5^ Immunogenicity of the local radiation therapy treatment is important for combination with immunotherapy, such as immune checkpoint inhibitors.^6^ Vanpouille-Box et al.^7^ demonstrated radiation-induced activation of the cGAS/STING pathway with a subsequent type-I-interferon response, initially triggered by the occurrence of dsDNA fragments in the cytoplasm. Additionally, the authors found a discontinuous and nonlinear dose response, where the balance between the presence of dsDNA fragments and expression of *Trex1*, which clears the cytoplasm from the fragments, determines the effectiveness. The most effective dose range in that context was 8 to 12 Gy, and a fractionated 3 × 8 Gy regimen was superior to a single high dose of ≥20 Gy based on the same mechanistic interplay.^7^ In this study, we investigate the potential of CIRT in a murine triple-negative breast cancer model in terms of overcoming radioresistance and especially in triggering immunogenicity via the occurrence of cytoplasmic dsDNA fragments and a subsequent type-I-interferon response.

## Material and Methods

### Cell lines and reagents

The mammary carcinoma cell line 4T1 was purchased from ATCC (CRL-2539) and cultured in Roswell Park Memorial Institute medium (RPMI 1640 + GlutaMAX-I, Gibco) supplemented with 10% fetal bovine serum (Sigma-Aldrich) and 1% penicillin streptomycin (10000 Units/mL Penicillin, 10 mg/mL Streptomycin, Gibco). The TS/A mammary adenocarcinoma cell line was purchased from EMD Millipore (SCC177) and cultured in Dulbecco’s Modified Eagle’s Medium (+ GlutaMAX^T^-I, Gibco) with high glucose containing 10% fetal bovine serum (Sigma-Aldrich) and 1% penicillin streptomycin (10,000 Units/mL penicillin, 10 mg/mL streptomycin, Gibco). All cell lines were maintained in culture with 20% O_2_, 5% CO_2_ at 37°C independent of the experiment and routinely screened for *Mycoplasma* [polymerase chain reaction (PCR) Mycoplasma test kit, ITW Reagents]. For some of the experiments, the cGAS inhibitor (RU.521, InvivoGen) was used at 20 µM, and the STING inhibitor (H-151, InvivoGen) at 1.5 µM. Both were added immediately before irradiation.

### Irradiation

Cells were seeded in 25 cm^2^ tissue culture flasks and at 70%-80% of confluency were exposed to X-ray. For quantifying cytoplasmic dsDNA, 7.5 × 10^5^ 4T1 cells per 3.5 cm Petri dishes were seeded 24 hours before irradiation. For radiograph irradiation, we used the vertical irradiation cabinet [radiograph generator Isovolt DSI (Seifert, Ahrensberg), 7 mm beryllium, 1 mm aluminum, and 1 mm copper filter system (250 kV/16 mA)] at a dose rate of 2 Gy/min at the GSI Helmholtz Center for Heavy Ion Research (Darmstadt, Germany). Carbon-ion irradiation (^12^C^6+^) was performed at the Marburg Ion Beam Therapy center (MIT, Marburg, Germany, MIT-2022-03) or at the SIS18 at the GSI Helmholtz Center for Heavy Ion Research, irradiating 5 × 10^6^ cells per dose (220 MeV/u; dose-averaged linear energy transfer of 75 keV/μm, 4 cm Spread Out Bragg Peak, SOBP) at a dose rate of about 2 Gy/min. Immediately after irradiation, the cells were seeded in 25 cm^2^ tissue culture flasks. For both radiation types, physical doses from 2 to 24 Gy were delivered. During irradiation, the cells were at room temperature and after radiation exposure they were immediately brought back to the previous culture conditions (20% O_2_, 5% CO_2_ at 37°C). For carbon-ion irradiation, some endpoints were performed only at doses of 2 Gy, 8 Gy, and 20 Gy, because of limitations in beam availability. Additionally, a fractionation regimen of 8 Gy given in 3 consecutive days (3 × 8 Gy) was performed for X-ray.

### Clonogenic cell survival

Clonogenic survival of 4T1 cells was assessed using the colony-forming assay as described in.^8^ After irradiation, cells were counted and plated in triplicates into 25 cm^2^ tissue culture flasks. The appropriate number of cells was seeded aiming at the statistically significant formation of 100 colonies. The cells were fixed and stained with methylene blue solution after 1 week of incubation. A colony was counted if composed of at least 50 cells.

### Immunofluorescence, confocal microscopy, and quantification of cytosolic dsDNA

For immunofluorescence staining, cells were fixed in 2% formaldehyde and permeabilized with 0.1% Triton X-100 for 10 minutes and blocked with 0.4% Bovine serum albumin in phosphate-buffered saline for 20 minutes. All antibodies were diluted in 1× phosphate-buffered saline, 0.4% Bovine serum albumin: αdsDNA (host: mouse; Abcam, ab27156) was used at a concentration of 1 µg/mL and αmouse Alexa Fluor 488 (host: goat; Invitrogen, A-11017) at a concentration of 5 µg/mL. To visualize the cytoplasmic area, we stained F-actin with Phalloidin-iFluor 647 Conjugate (Cayman, 20555). DNA was counterstained with 4′,6-diamidino-2-phenylindole (DAPI) (1 µg/mL, AppliChem, A1001). On mounting in Slow Fade Diamond Antifade (Invitrogen, S36963) the cells were imaged at a Nikon Eclipse Ti microscope. The exposure time for the immunostained dsDNA signal was optimized for the cytosolic signal, which in most cases caused overexposure of the nuclear dsDNA signal. To quantify the cytosolic dsDNA signals within the images, we used the in-house developed software ImageD (versions v1_7_0 and v2_0_4, plugin “cytoplasmic dsDNA detection” https://github.com/DavidEilenstein/ImageD). With this software, dsDNA signals ≥5 px were quantified as cytoplasmic dsDNA foci, if they were located outside the DAPI-stained area (nucleus) and inside the phalloidin-stained area (cytoplasm). dsDNA foci counted in 0 Gy samples (background) were subtracted from dsDNA foci numbers at doses >0 Gy. For X-ray, 3 to 6 independent experiments were performed (exception: 2 Gy; 1 experiment). For carbon-ions, 2 independent experiments were performed (exception: 20 Gy; 1 experiment). Per experiment, at least 53 cells (20 Gy X-ray) and up to 1425 cells (0 Gy carbon ions) were analyzed per dose.

The cytoplasmic dsDNA after radiograph exposure was additionally quantified from 2 independent experiments with the AccuClear nano dsDNA kit (Molecular Devices, R8357). Cytoplasmic extracts were obtained according to^9^ and the results were collected with a SpectraMax i3x reader.

### Gene expression

Total RNA from cell pellet was extracted with RNeasy Mini Kit (Qiagen) according to the manufacturer’s instructions, including a DNase digestion step (RNase-free DNase set, Qiagen). To perform cDNA synthesis, 2 µg of extracted RNA was used, according to the manufacturer’s instructions (RevertAid First Strand cDNA Synthesis Kit, Thermo Fisher Scientific). Subsequently, the cDNA was loaded in a quantitative PCR plate (Sarstedt) with Fast SYBR Green Master Mix (Thermo Fisher Scientific) and bioinformatically validated specific primers (Gapdh, QT00199388; Rpl13a, QT00267197; Ifnb1, QT00249662; Trex1, QT00288967; QuantiTect Primer Assay, Qiagen). The final volume was 20 µL and the cycling conditions were a holding stage of 95°C for 15 minutes, followed by 40 cycles of denaturation at 95°C for 15 seconds, annealing at 60°C for 30 seconds, extension at 72°C for 20 seconds. A melt curve analysis was performed to ensure the specificity of the amplicons. The quantitative PCRs were run using QuantStudio 3 system (Thermo Fisher Scientific) and the results were collected with QuantStudio Design & Analysis Software (version 1.5.2, Thermo Fisher Scientific). The fold change of the genes of interest was calculated with the 2^(–ΔΔCt)^ method, normalizing the *Cq* values for the geometric mean of 2 reference genes (*Gapdh, Rpl13a*). The stability of the reference genes after irradiation is reported in Table E1.

### ELISAs

Interferon beta (IFN-β) release was quantified with ELISA kit (PBL Assay Science, PBL42410-1) according to manufacturer’s instructions. An additional dilution of the standard curve was necessary because of the low concentration of the analyte in the controls. For cyclic guanosine monophosphate-adenosine monophosphate (cGAMP) assessment, cells were pretreated with M-PER Mammalian Protein Extraction Reagent (Thermo Fisher Scientific) following manufacturer’s instructions, according to the sample preparation recommendations of the ELISA kit (2′3′ cGAMP ELISA kit, Cayman Chemical, 501700). The ELISA was performed according to manufacturer’s specifications. Absorbance (450 nm) was measured with BioTek EL808 Microplate Reader and Gen5 (version1.11) software.

### Bulk RNA sequencing

Sequencing was performed by Arraystar Inc. In brief, 1-2 µg of total RNA of 4T1 and TS/A cells were used to prepare the sequencing library. The libraries were sequenced on Illumina NovaSeq 6000 instrument. Fold change (cutoff 1.5), *P* value (≤.05), and FPKM (≥.5 mean in one group) were used for filtering differentially expressed genes and transcripts. The *Z*-score was calculated for some Genes Of Interest. For further details, see the supplementary section.

### Statistics

Unless otherwise specified, the experiments were performed in 3 biological replicates for X-ray and carbon ions, and data are presented as mean ± SD. The data were analyzed with GraphPad Prism software (version 9.3.1) and statistically significant differences between X-ray and carbon ions were determined using unpaired 2-tailed *t*-test or 2-way analysis of variance with Tukey’s multiple comparisons test and considered significant for *P* values ≤.05, unless indicated elsewise. For further information on the performed fits, we refer to the supplementary section.

## Results

### Carbon ions are more effective than X-ray in killing triple-negative breast cancer cells and in the induction rate of cytoplasmic dsDNA foci

**Figure 1:**
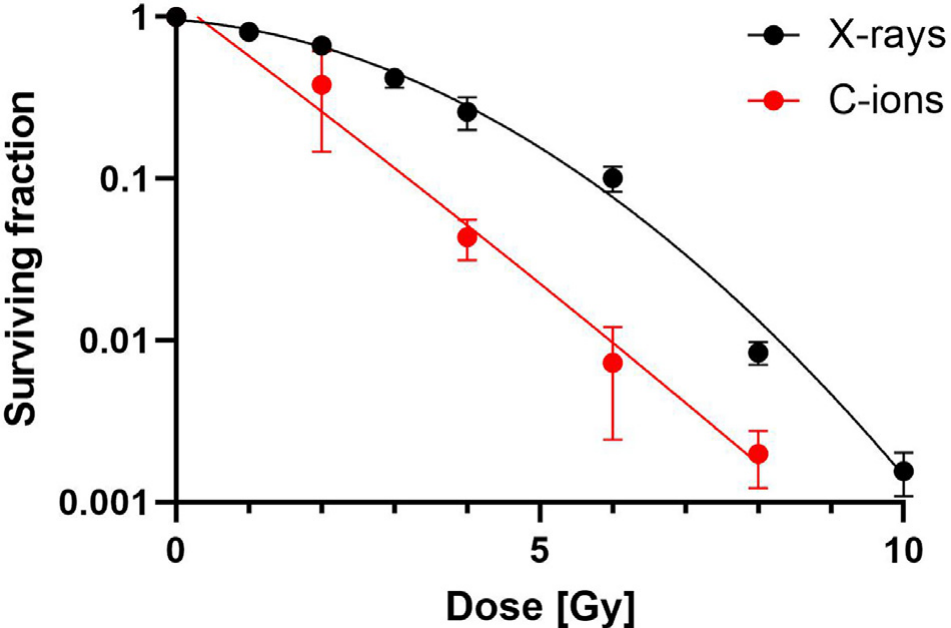
Reduced clonogenic cell survival after carbon-ion exposure. Colonies of surviving 4T1 cells were counted after exposure to X-ray or carbon ions and plotted as surviving fraction. Curves were fitted using the linear quadratic model. The relative biological efficiency was calculated to be roughly 2 at a level of 10% survival. Data for X-ray were derived from Reppingen et al.^24^

**Figure 2:**
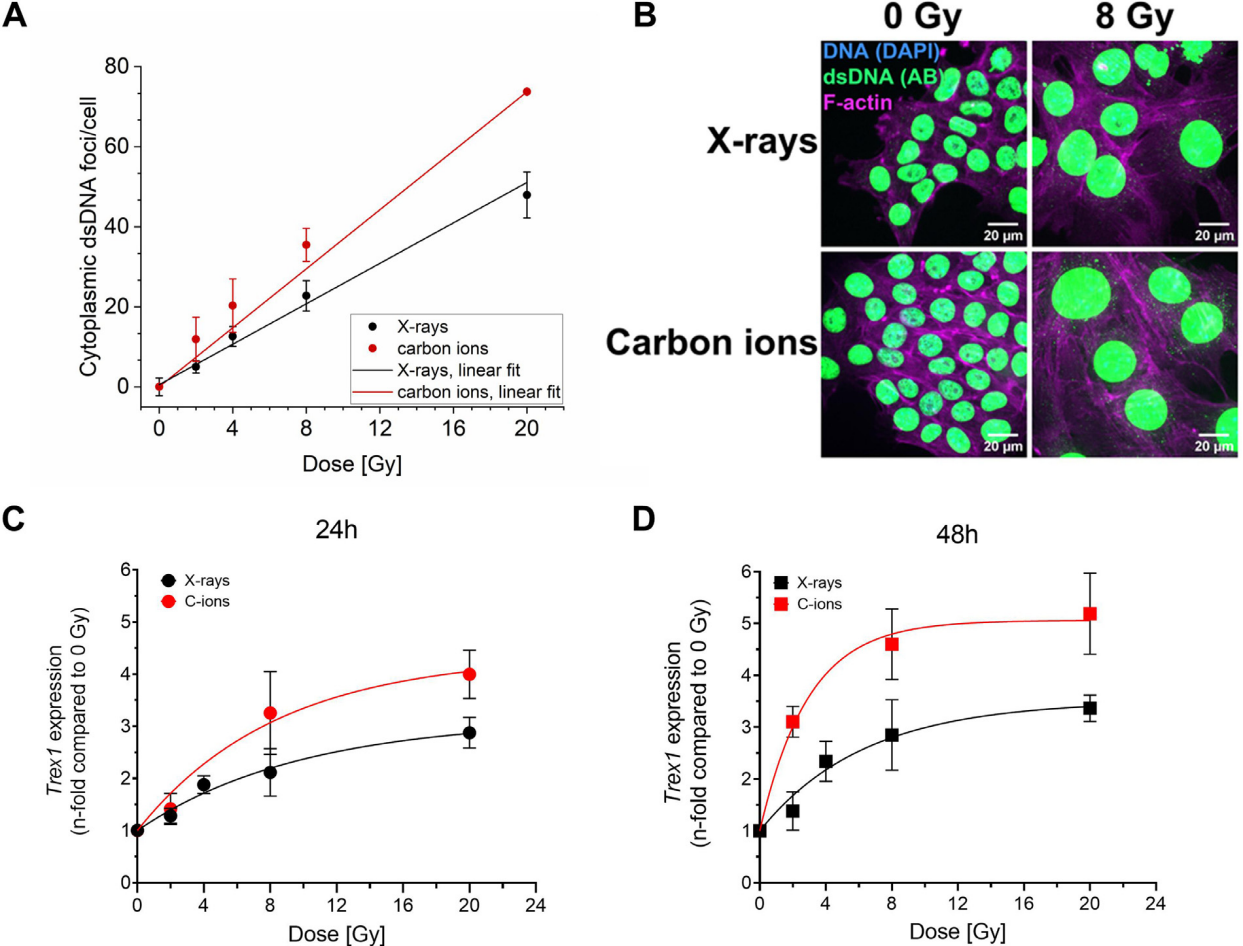
Carbon-ion irradiation features a higher rate of foci of cytoplasmic dsDNA fragments and a higher expression of *Trex1*. (A) Foci of dsDNA fragments per cell in dependence of the dose. Numbers of cytoplasmic dsDNA foci of nonirradiated cells were subtracted. 4T1 cells were irradiated with X-ray or carbon ions. Carbon ions resulted in a significantly higher rate of foci induction with increasing doses 24 hours after irradiation (*P* < .0001, *t*-test comparing the slopes of the 2 fits; see supplement for details on the statistical analysis). (B) Representative images of 4T1 cells 24 hours after 0 Gy or 8 Gy radiograph or carbon-ion irradiation. dsDNA was immunofluorescence stained (AB), F-actin as a cytosol marker was stained with phalloidin, and DNA was also counterstained with DAPI. dsDNA signals outside the DAPI-stained nucleus but inside the phalloidin-stained area were counted as cytoplasmic dsDNA foci. Expression of *Trex1* in 4T1 cells increases with dose at 24 hours (C) and 48 hours (D). The curves tend to reach a plateau at high doses. Comparison of the trends was performed using an exponential function (see Supplements). The difference between X-ray and C-ions is not statistically significant at 24 hours but is statistically significant at 48 hours comparing the parameters of the fits (see Supplementary materials). The difference comparing the radiation qualities at each dose is statistically significant after 24 hours at 20 Gy (*P* = .024) and after 48 hours at all doses (*P* = .0032 at 2 Gy, *P* = .0345 at 8 Gy, and *P* = .0186 at 20 Gy; 2-tailed t-test).

Carbon ions (C-ions) are more effective than X-ray in 4T1 cell killing and the relative biological effectiveness is about 2 (Fig. 1). We then measured the induction of dsDNA in the cytoplasm after radiation (Figs. 2A, B, E1 and E2). Carbon ions resulted in a significantly higher rate of cytoplasmic dsDNA foci per cell when compared to photons 24 hours after exposure (Fig. 2A, B). We also investigated the dose response of *Trex1* expression, which is known to remove dsDNA fragments from the cytoplasm. Expression of *Trex1* increased continuously with dose at 24 hours from radiograph exposure, and slightly higher values were observed after carbon ions at high doses compared to the same radiograph doses (Fig. 2C). Expression of *Trex1* remained similar both for amplitude and dose response at 48 hours (Fig. 2D). To corroborate such dose response, we investigated the *Trex1* expression in TS/A cells, confirming the increase with dose (Fig. E3A, B). Overall, we found a linear dose-dependent increase in cytoplasmic dsDNA foci, whereas *Trex1* levels reached a plateau at high doses and seemed not to have a threshold dose from which on its expression would strongly increase to remove the dsDNA fragments. To link these observations with the activity of cGAS, we measured the concentration of cGAMP (a product of cGAS activation) at 24 hours after exposure to photons or carbon ions (Fig. E4). It slightly increased with dose with carbon ions being in tendency (not significantly) more efficient.

### Carbon-ion irradiation results in an elevated interferon-β release indicating its potential for increased immunogenicity

**Figure 3:**
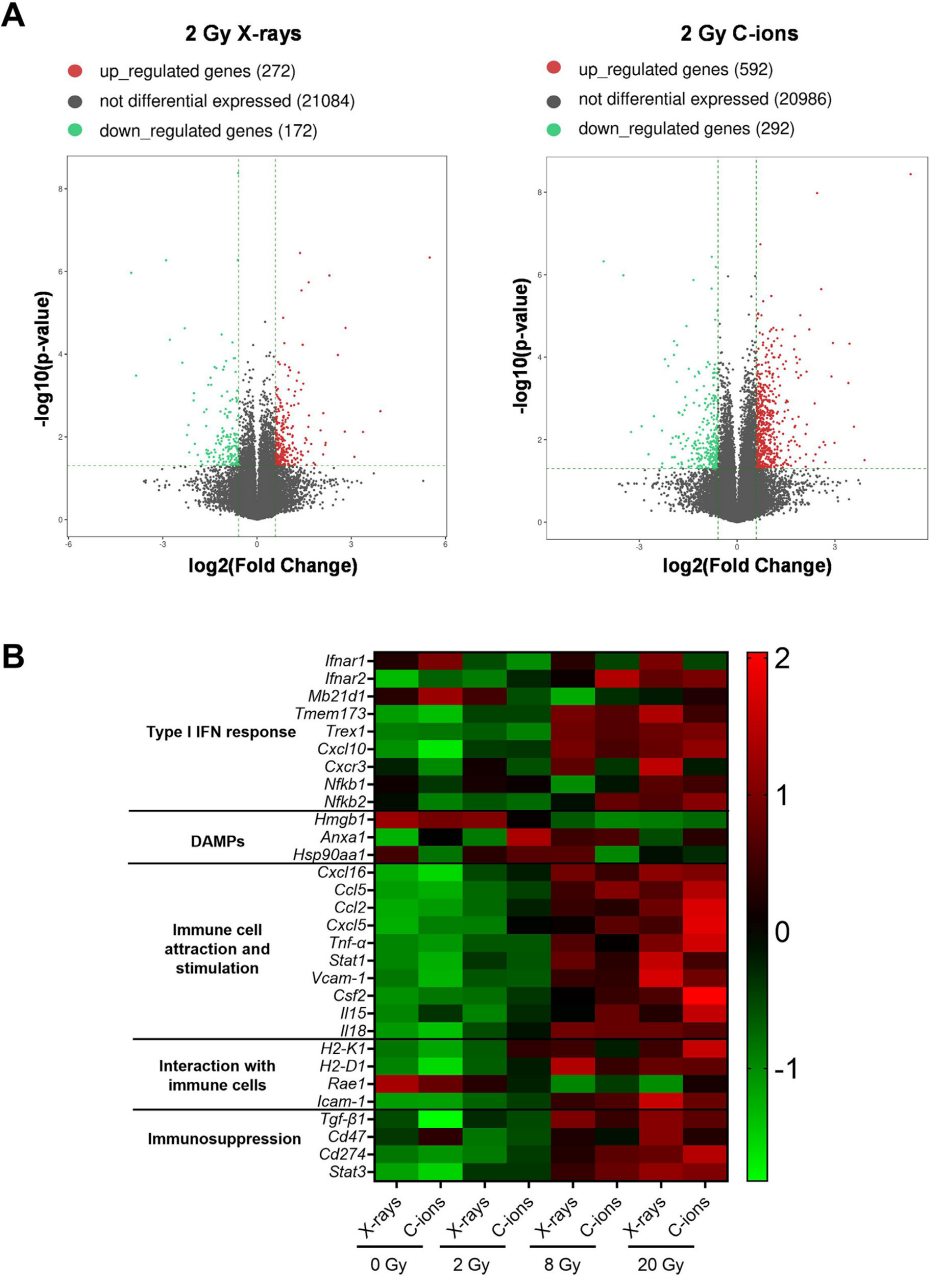
At high doses, both X-ray and carbon ions upregulate genes involved in immunogenicity in 4T1 cells. Volcano plot of differentially expressed genes at 24 hours after 2 Gy irradiation with X-ray (left) or carbon ions (right) compared to the respective nonirradiated samples (0 Gy). Significantly upregulated genes and downregulated genes are depicted in red and green, respectively, and nonsignificant genes are shown in gray (A). Heat map visualization of the *Z*-score of some genes of interest (rows) across different doses and radiation qualities (columns, see supplements for details). Colors represent relative expression levels among the doses and radiation types, with green for low expression and red for high expression (B).

We performed bulk RNA sequencing 24 hours after exposure to X-ray or carbon ions to study changes in gene expression based on the different radiation qualities (Fig. 3). At doses of 2 Gy and 8 Gy, carbon ions led to a higher number of genes differentially expressed than X-ray (Figs. 3A, and E5A) when compared to their respective controls. Instead, at a dose of 20 Gy, similar numbers of differentially expressed genes are observed comparing the 2 radiation qualities (Fig. E5B). Figure 3B displays a set of selected genes related to immunogenic signaling. Generally, the expression of these genes increases after 8 or 20 Gy of X-ray or carbon ions, with a slightly higher expression for 20 Gy carbon ions in some of the genes. This was found both for genes related to immunoactivation and immunosuppression and confirmed in TS/A cells on exposure to X-ray when compared to 4T1 cells (Fig. E6A, B). As for damage-associated molecular patterns, no coherent pattern of dose response was found. More in detail, focusing on genes related to the type-I-interferon response, *Ifnb1* expression was below the limit of quantification with this method at 24 hours and is therefore not displayed here. *Trex1* expression pattern over dose confirmed the dose response found by quantitative reverse transcription PCR (see Fig. 2). Similar dose responses were also found for *Cxcl10*, a gene coding for a cytokine (CXCL10) released on auto- and paracrine type-I-interferon signaling. Interestingly, *Cxcr3*, a gene for a receptor of CXCL10 appeared not to be increased after carbon-ion exposure.

**Figure 4:**
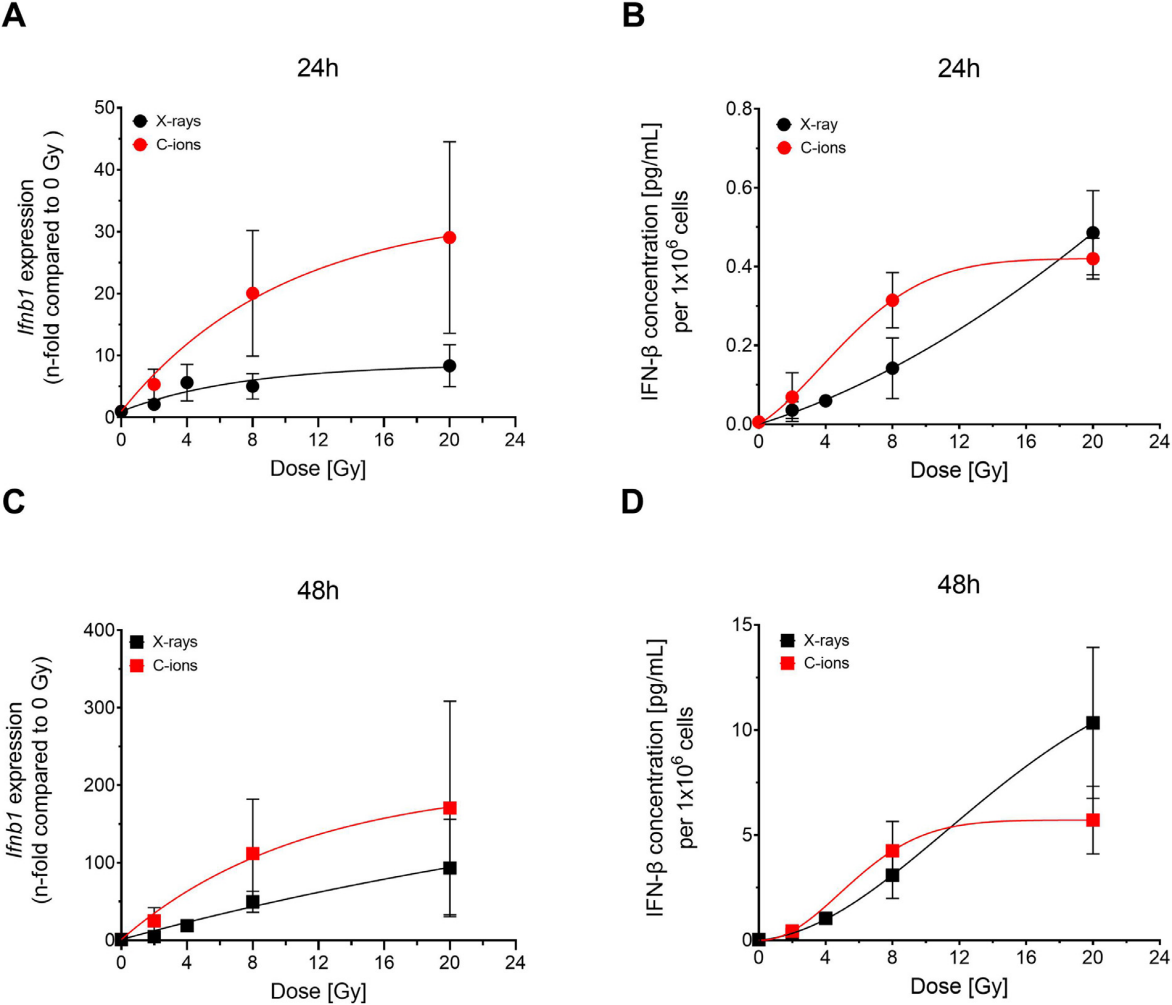
Expression and release of interferon-β increase with dose after X-ray or carbon ions. Expression of *Ifnb1* was measured at 24 hours (A) and 48 hours (C) after exposure to X-ray or carbon ions. At similar time points, the release of IFN-β (B and D) was assessed. With the exception of 20 Gy in release, where a plateau was reached, carbon ions were more efficient in tendency, but not significantly with respect to the fit of the functions (see Supplementary material) in 4T1 cells. The difference in *Ifnb1* expression comparing the 2 radiation qualities at each dose is not statistically significant at any dose and time point (2-tailed *t*-test). The difference in IFN-β release comparing the 2 radiation qualities at each dose is statistically significant only 24 hours after irradiation with 8 Gy (*P* = 0.0459, 2-tailed *t*-test).

Because IFN-β is a crucial factor in the type-I-interferon response, yet was not detectable in bulk RNA sequencing, we measured the expression of *Ifnb1* with quantitative reverse transcription PCR and the release of IFN-β at 24 hours after exposure. *Ifnb1* gene expression showed an increase with dose and carbon ions being more effective than photons (Fig. 4A). Although the dose-response patterns differ for *Ifnb1* expression and the occurrence of cytoplasmic dsDNA (Fig. 2A), both endpoints show an increase with dose and a higher efficiency of carbon ions. The release of IFN-β at 24 hours was generally low with little differences between the 2 radiation types (Fig. 4B). Therefore, we investigated both expression and release also 48 hours after exposure (Fig. 4C, D). The levels of both were elevated with respect to 24 hours and the dose response stayed similar. Comparable dose responses after radiograph exposure were again confirmed in TS/A cells (Fig. E3C-F).

To demonstrate the link of the effects to the activation of the cGAS/STING pathway, we next investigated its downstream signal, that is, the expression of the *Ifnb1* gene after the inhibition of each cGAS or STING. Although exposure to X-ray resulted in an increased expression of *Ifnb1* at 24 hours, application of cGAS or STING inhibitors diminished (cGAS inhibitor, Fig. E7A) or eradicated (STING inhibitor, Fig. E7B) the effect, demonstrating the involvement of the cGAS/STING pathway.

**Figure 5:**
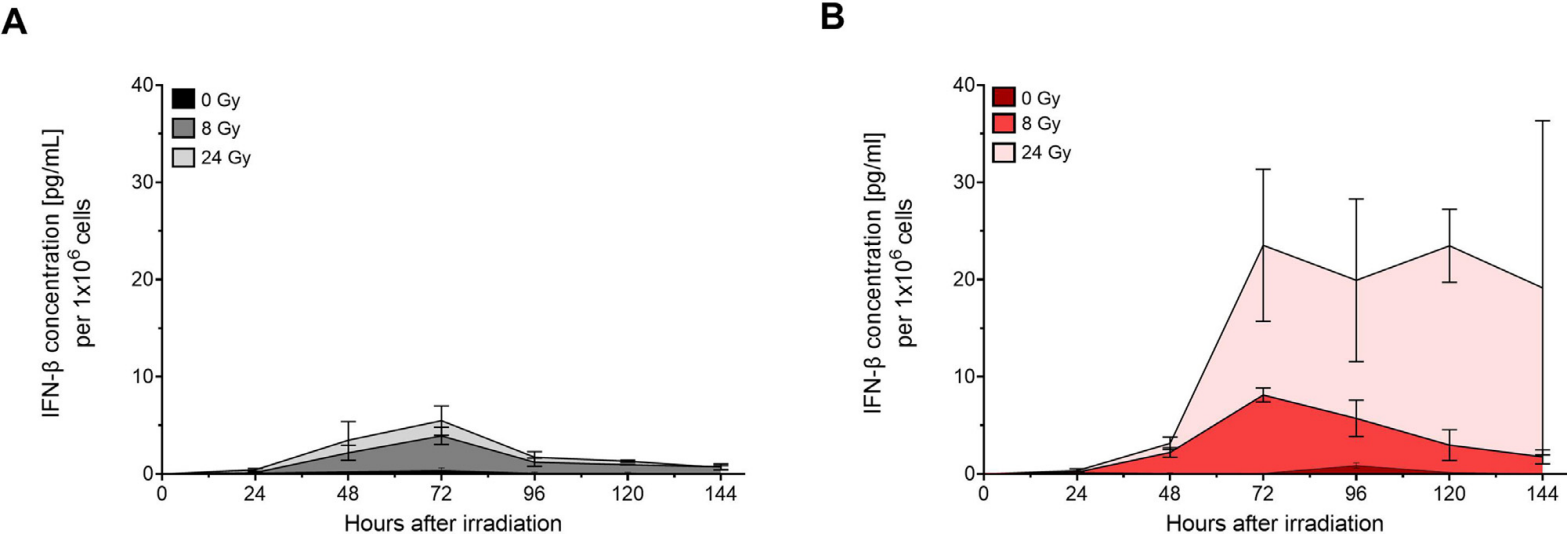
Carbon ions induce a higher release of interferon-β over time. The release of IFN-β was measured up to 144 hours after exposure to 8 Gy or 24 Gy X-ray (A) or carbon ions (B) in 4T1 cells. At 72 hours post irradiation, carbon ions cause a significantly higher release of IFN-β than X-ray (8 Gy *P* = .0004, 24 Gy *P* = .0019, unpaired 2-tailed *t*-test, N=5 X-ray, N=3 carbon ions). *Abbreviation*: IFN-β, interferon beta.

Based on the observation that the expression of *Ifnb1* and the release of its gene product is higher 48 hours than 24 hours after irradiation, we speculated that a differential response in IFN-β release might occur only at later time points. Therefore, we measured the amount of released IFN-β in dedicated samples daily on a time course of up to 144 hours (Fig. 5A, B). Indeed, at 72 hours after irradiation, we detected strong differences (*P* = .0004 and *P* = .0019 for 8 Gy and 24 Gy, respectively) in the release of IFN-β between photons and carbon ions. The release was not only stronger but also more persistent after exposure to carbon ions (Fig. 5B), indicating an increased potential for a robust immunogenic signal.

### A single high dose is comparatively effective to a fractionated scheme after exposure to photons with respect to IFN-β release

**Figure 6:**
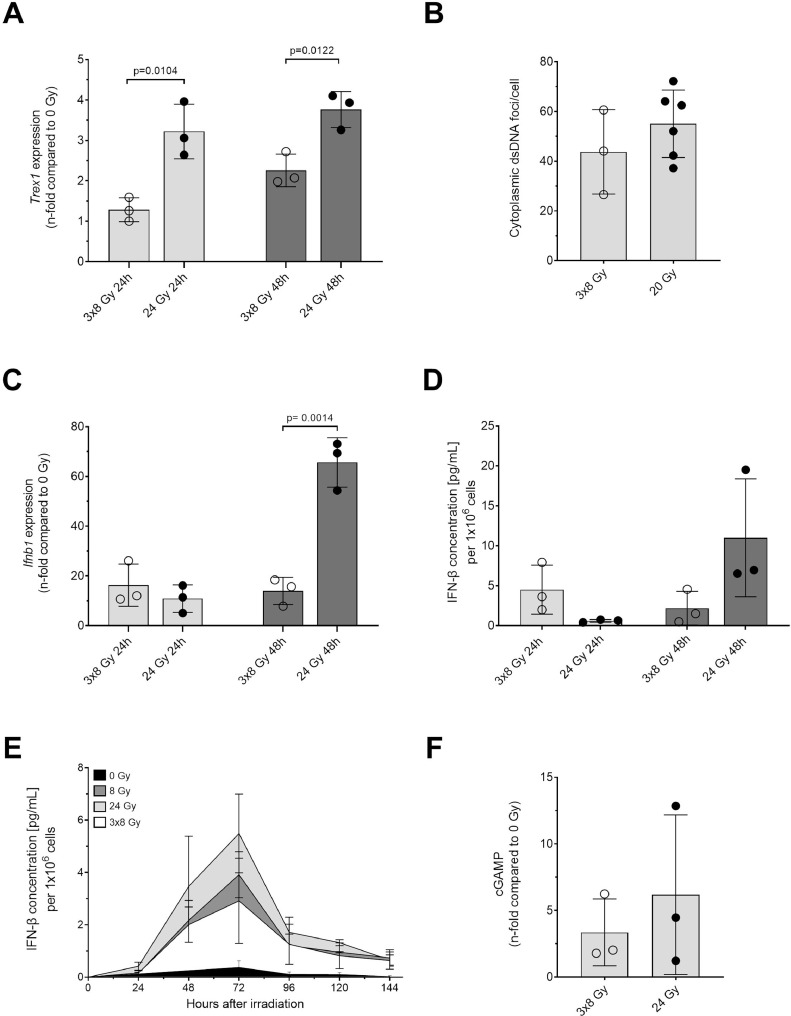
Single high doses and a 3 × 8 Gy hypofractionation scheme similarly induce the release of interferon-β in 4T1 cells. The endpoints tested for the dose-response curves were also performed by comparing a single high dose of 20 or 24 Gy to a 3 × 8 Gy hypofractionation scheme at 24 hours or 48 hours after exposure to X-ray. More in detail, *Trex1* expression (A), cytoplasmic dsDNA foci 24 hours after X-ray (B), *Ifnb1* expression (C), IFN-β release (D), IFN-β release over time (E) and cGAMP 24 hours after X-ray (F) were measured. Significances were tested using an unpaired 2-tailed *t*-test. (E) Data for single doses are derived from Fig. 5A. *Abbreviation*: IFN-β, interferon beta.

We investigated whether a hypofractionated regimen of 3 × 8 Gy was superior to a single high dose of photons (24 Gy) (Fig. 6A-F). Our results do not support the hypothesis that fractionation increases cGAS-STING pathway activation. Although we indeed found a reduced level of *Trex1* expression at 24 hours and 48 hours after exposure to 3 × 8 Gy (Fig. 6A), this did not translate in higher amounts of cytoplasmic dsDNA foci per cell (Fig. 6B). Although slightly higher amounts of *Ifnb1* expression and IFN-β release were measured at 24 hours after exposure to 3 × 8 Gy X-ray, the effect did not persist because at 48 hours after exposure the effect was found inversed (Fig. 6C, D). This is confirmed by IFN-β release over time, in which following 3 × 8 Gy there is a lower release than after a single dose of 24 Gy (Fig. 6E). Measuring cGAMP as a surrogate of cGAS activation, again, we found a similar effect (Fig. 6F). Measuring *Trex1* and *Ifnb1* expression as well as IFN-β release in TS/A cells confirmed little to no differences between 24 Gy and 3 × 8 Gy (Fig. E8A-C).

## Discussion

CIRT is increasingly used worldwide because it features improved precision in beam delivery and an increased biological effectiveness, which allows the treatment of radioresistant tumors with close vicinity to sensitive organs.^10^ Additionally, CIRT is considered to feature an increased immunogenicity, which renders it a good match for immunotherapies and the treatment of metastatic cancer disease.^11^, ^12^, ^13^ Clinical trials are currently being set up to prove this hypothesis.^14^^,^^15^ Using the poorly immunogenic murine breast cancer model 4T1,^16^ we investigated the immunogenicity of carbon ions with respect to the type-I-interferon response triggered by the cGAS/STING axis, which in turn is activated by the presence of dsDNA foci in the cytoplasm.^17^

We hypothesized that carbon ions can be more effective with respect to the occurrence of cytoplasmic dsDNA fragments because of their peculiar features of DNA damage induction.^18^ Indeed, our data indicate that carbon ions resulted in an increased rate of cytoplasmic dsDNA foci induction as radiation dose increases. In addition, we show an increased release of IFN-β after carbon-ion exposure, pointing to a higher potential for a strong type-I-interferon response as compared to X-ray. The experiments inhibiting cGAS and STING indicate that, although STING plays a central role in radiation-induced type-I-interferon response, cGAS may not exclusively contribute to STING activation after cytoplasmic dsDNA detection. This implies that other sensors might play a role in type-I-interferon activation after irradiation and it remains to be elucidated whether they are differentially induced by carbon ions.

Furthermore, the increased efficiency of carbon ions with respect to clonogenic cell survival, hence overcoming radioresistance of a triple-negative breast cancer model, provides additional rationale for the application of CIRT in breast cancer therapy.^4^ In this context, the dual role of interferons has to be considered because a sustained or prolonged IFN signaling may result in adverse effects related to therapy resistance.^19^^,^^20^ Of note, our data indicated lower expression of the gene coding for CXCR3 for carbon ions as compared to X-ray. Coexpression of CXCL10/CXCR3 on tumors was reported to be adverse with respect to metastasis development and to be a marker for poor clinical outcome. Autocrine signaling of the Cxcl10/Cxcr3 axis was shown to promote tumor cell growth, motility, and metastasis.^21^ Interestingly, a reduced metastatic potential for CIRT has already been reported in other preclinical models.^12^^,^^22^

We failed to reproduce the previously reported discontinuous dose response, favoring a single dose in the range of 8 to 12 Gy over a single high dose because of the mechanistic interplay between the occurrence of cytoplasmic dsDNA fragments and *Trex1* expression.^7^ In both murine breast cancer models, 4T1 and TS/A cells, used as well by Vanpouille-Box et al., our data show an increase of the type-I-interferon signal with dose throughout several endpoints. The former also reported the best responses for a 3 × 8 Gy regimen as compared to single doses, again based on the above-mentioned mechanistic dualism. Our study instead resulted in comparable signals for a single dose of 24 Gy versus a 3 × 8 Gy regimen. Despite using the same cell lines, different results between laboratories can be explained with the application of different materials such as fetal bovine serum or different assays. Moreover, the method of choice for quantification of cytoplasmic dsDNA in our study is based on the assessment of foci per cell, rather than recovering a total signal in a group of cells, which could partly account for the differences.

Nonetheless, we found a coherent increase with dose in all endpoints. Vanpouille-Box et al. showed that their results translated in vivo when the respective doses were applied in a mouse model in combination with immune checkpoint inhibitors, corroborating their hypothesis of superiority of a fractionated regimen over a single high dose.^7^ Our study lacks an in vivo translation as of now, which could verify this dose response and the superiority of CIRT over photons. Our data also indicate the importance of assessing several time points and later than the commonly used 24 hours after exposure in in vitro studies, as a differential release of cytokines occurred only at later time points, likely related to the radiation-induced cell cycle block.

If verified in further preclinical models, our results would argue for the application of higher doses than the classical 2 Gy fractionation scheme, because a dose of 8 Gy consistently resulted in enhanced signals. Also, despite not being superior to high doses of ≥20 Gy, we found a 3 × 8 Gy scheme at least comparable to 8 Gy. In fact, hypofractionated schemes applying 3 × 8 Gy or schemes in that range are increasingly used in clinical settings.^23^ Because carbon ions mostly performed better than X-ray at 8 Gy, our data encourage the application of 8 Gy or hypofractionated regimens also for CIRT, and indeed clinical studies with such regimens are already set up.^14^

Finally, because the occurrence of dsDNA fragments in the cytoplasm is the base of the type-I-interferon response, and because carbon ions resulted in a higher amount of dsDNA foci, the question of by which mechanism the fragments travel to the cytoplasm and how can that be differentially affected through carbon ions^18^ becomes crucial but remains to be addressed. In summary, our data support the application of CIRT in the context of immunotherapy combinations but underpin that further research is required at the same time to better exploit its potential advantages.

## Disclosures

Nicole B. Averbeck reports financial support was provided by German Federal Ministry of Economic Affairs and Climate Action. Claudia Fournier reports financial support was provided by German Federal Ministry of Education and Research. Burkhard Jakob reports financial support was provided by German Federal Ministry of Education and Research. If there are other authors, they declare that they have no known competing financial interests or personal relationships that could have appeared to influence the work reported in this paper.

## Appendix Supplementary materials

Supplement_2025-02-14 Image, application 1

## Footnotes

Supplementary material associated with this article can be found in the online version at doi:10.1016/j.adro.2025.101783.
