# Supplementary material for: Induction of Cytoplasmic dsDNA and cGAS-STING Immune Signaling After Exposure of Breast Cancer Cells to X-ray or High-Energetic Carbon Ions
Source: Adv Radiat Oncol. 2025 Apr 7;10(6):101783. doi: 10.1016/j.adro.2025.101783 (PMC12143610; doi:10.1016/j.adro.2025.101783)
Supplement: Supplement_2025-02-14 [file mmc1.docx]

**Supplement**

**
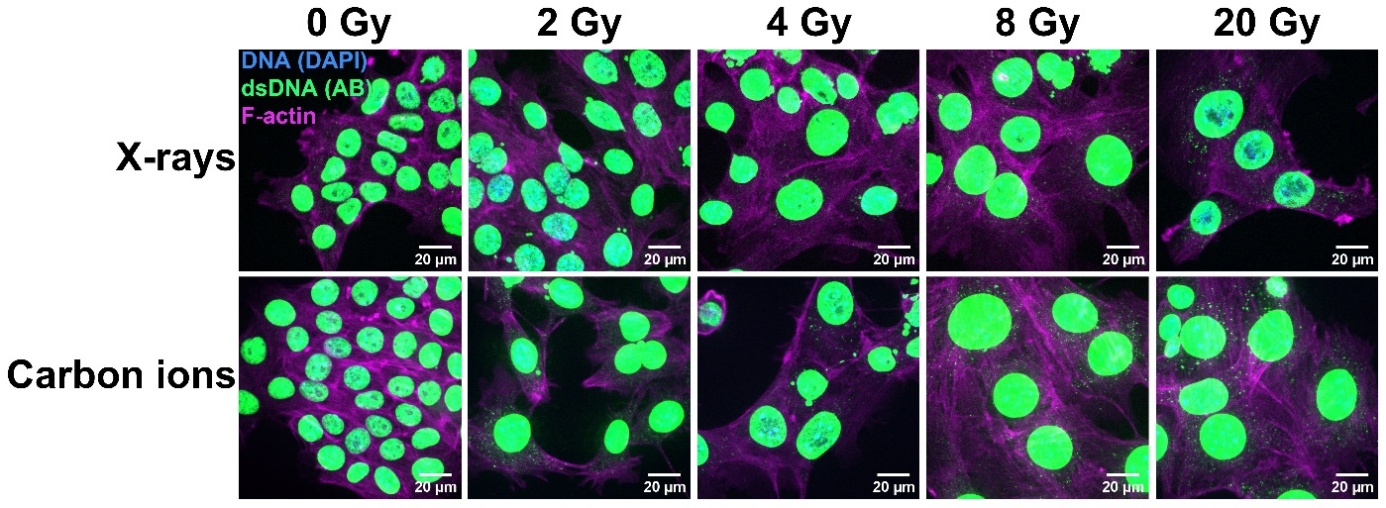
**

**Figure E1. Cytoplasmic dsDNA foci after irradiation with X-rays or carbon ions.** Representative images of 4T1 cells 24h after 0 Gy, 2 Gy, 4 Gy, 8 Gy, and 20 Gy X-ray or carbon ion irradiation. dsDNA was immunofluorescence stained (AB), F-actin as a cytosol marker was stained with phalloidin, and DNA was also counterstained with DAPI.

**
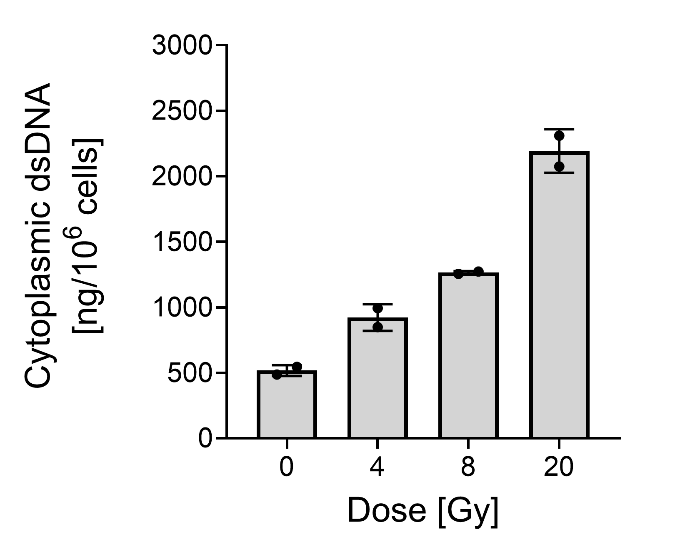
**

**Figure E2. Cytoplasmic dsDNA quantification from cytoplasmic extracts show a dose-dependent increase following X-ray exposure.** The cytoplasmic dsDNA was additionally quantified with the AccuClear nano dsDNA kit after X-rays. The results show a dose-dependent increase in cytoplasmic dsDNA comparably to the results obtained in Figure 2A.


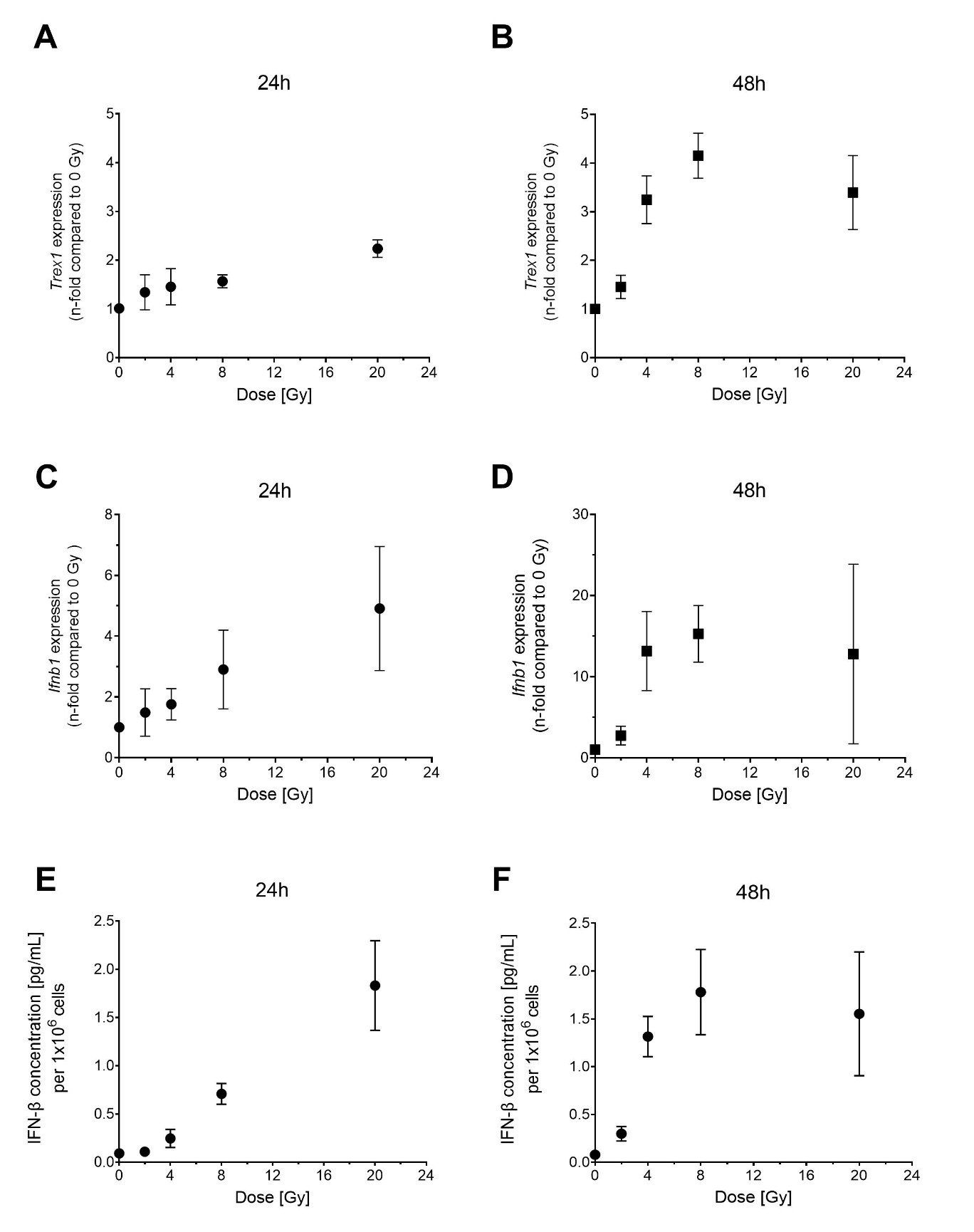


**Figure E3**. **Dose-dependent increase in the expression of *Trex1, Ifnb1,* and release of IFN-β in TS/A cells.** Expression of *Trex1* (A 24h, B 48h) and *Ifnb1* (C 24h, D 48h) as well as release of IFN-β (E 24h, F 48h) were measured following exposure to X-rays.


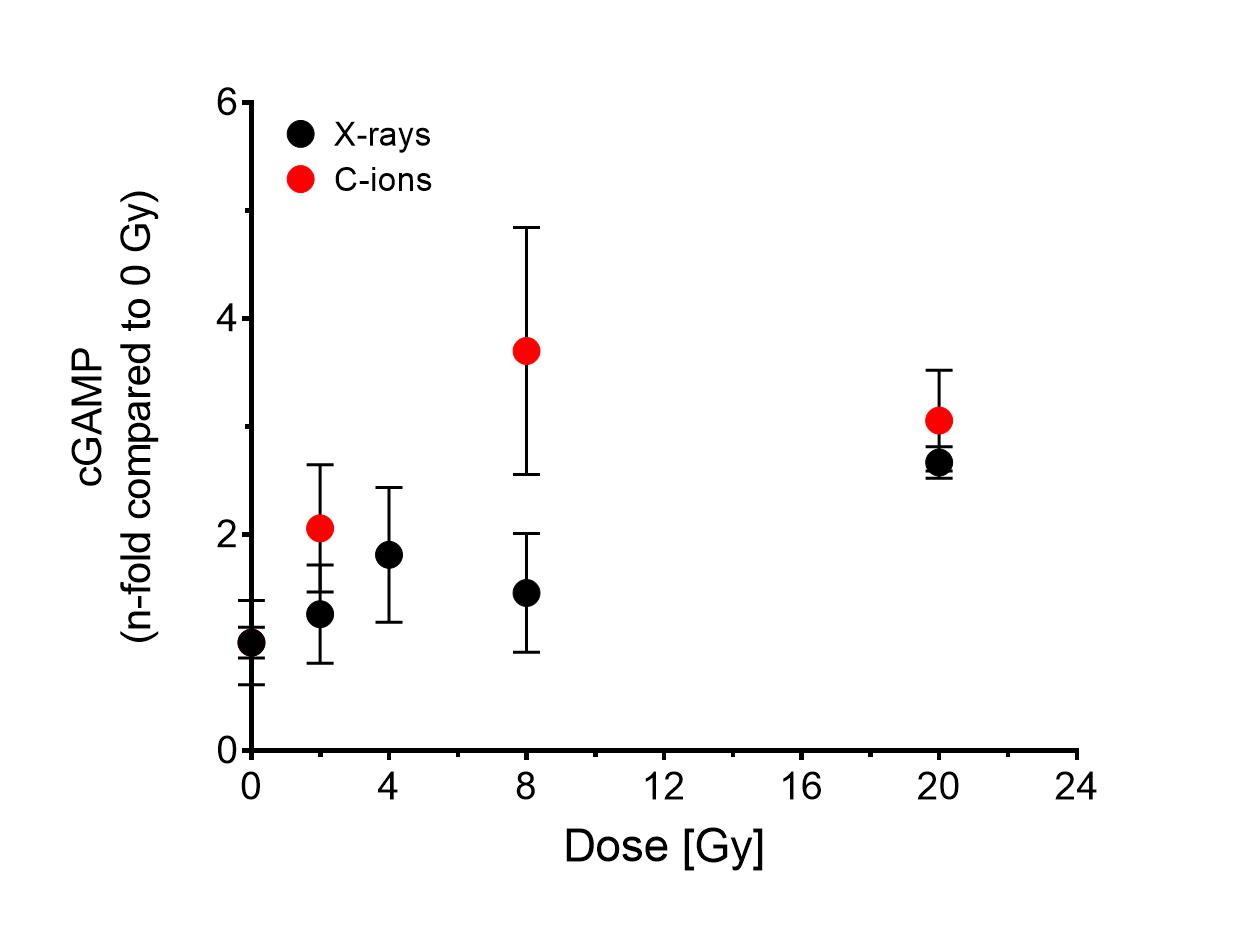


**Figure E4**. **cGAMP concentration slightly increases with dose in 4T1 cells.** The cellular concentrations of cGAMP (surrogate for cGAS activation) were measured 24h after exposure to X-rays or carbon ions. No significant differences were found between radiation qualities (unpaired two-tailed t-test).


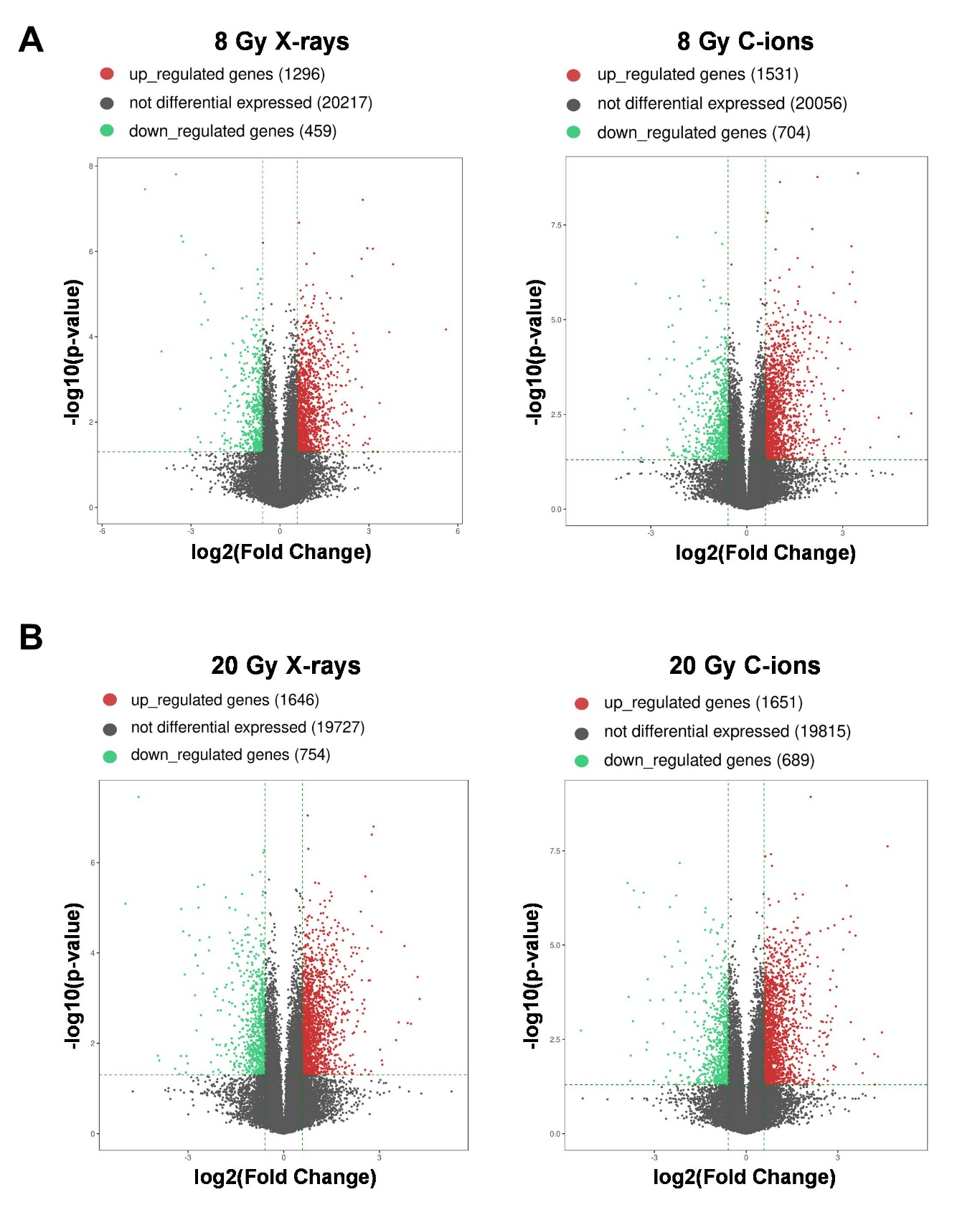


**Figure E5**. **The number of differentially expressed genes comparing high doses of X-rays and carbon ions is similar in 4T1 cells**. Volcano plots of differentially expressed genes 24h after 8 Gy (A) or 20 Gy (B) irradiation with X-rays (left) or carbon ions (right) compared to the respective non-irradiated samples (0 Gy). Significantly upregulated genes and downregulated genes are depicted in red and green, respectively, and non-significant genes are shown in gray. Increasing the dose, the number of differentially expressed genes becomes comparable between the two radiation qualities.


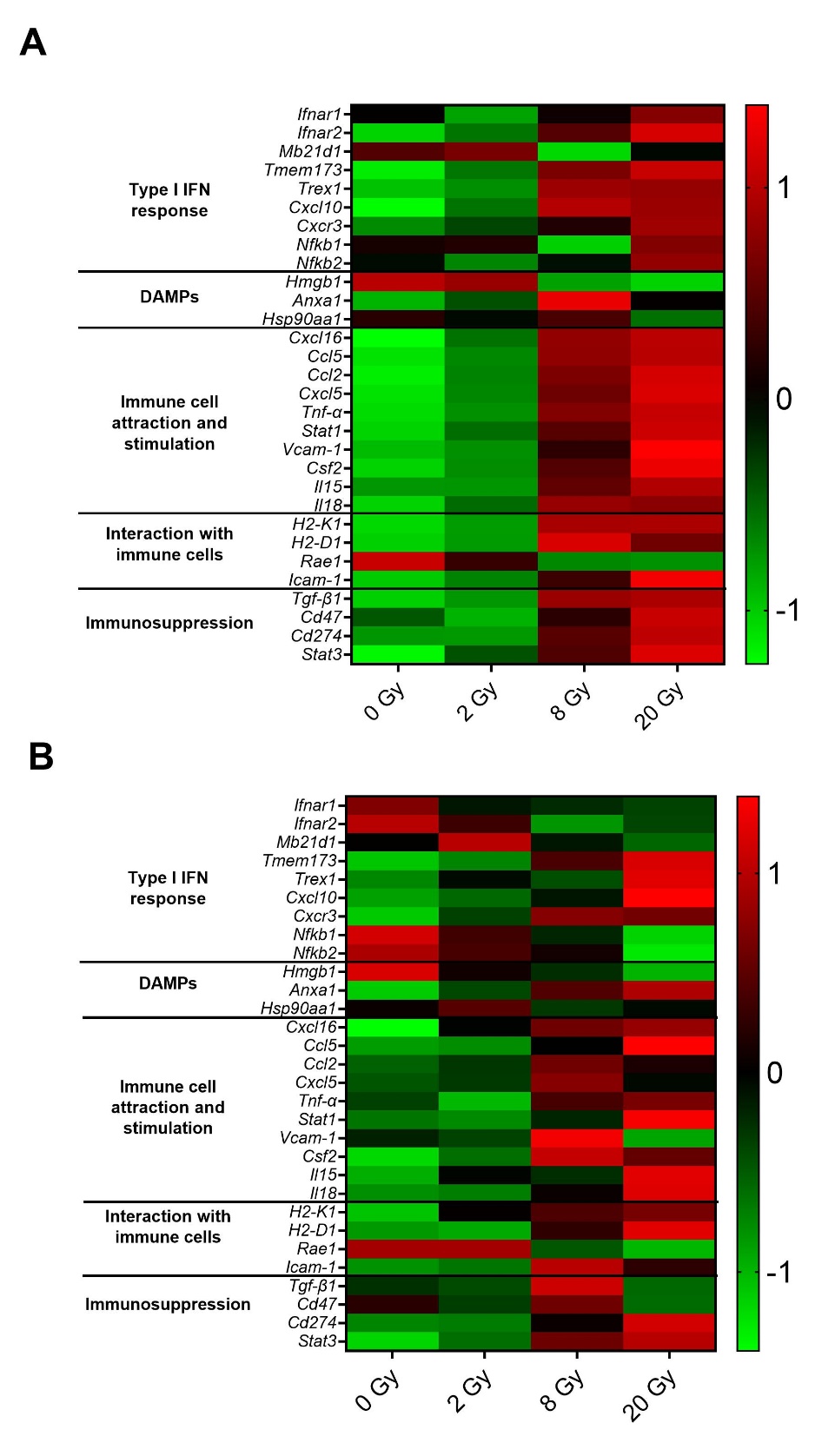


**Figure E6**. **Increased expression of selected genes after high doses of X-rays in 4T1 and TS/A cells.** The plot depicts the heat map of the Z-score of some genes of interest (rows) across different doses (columns, see Supplements for details) for 4T1 (A) and TS/A (B) cells. Colors represent relative expression levels amongst the doses, with green for low expression and red for high expression levels.


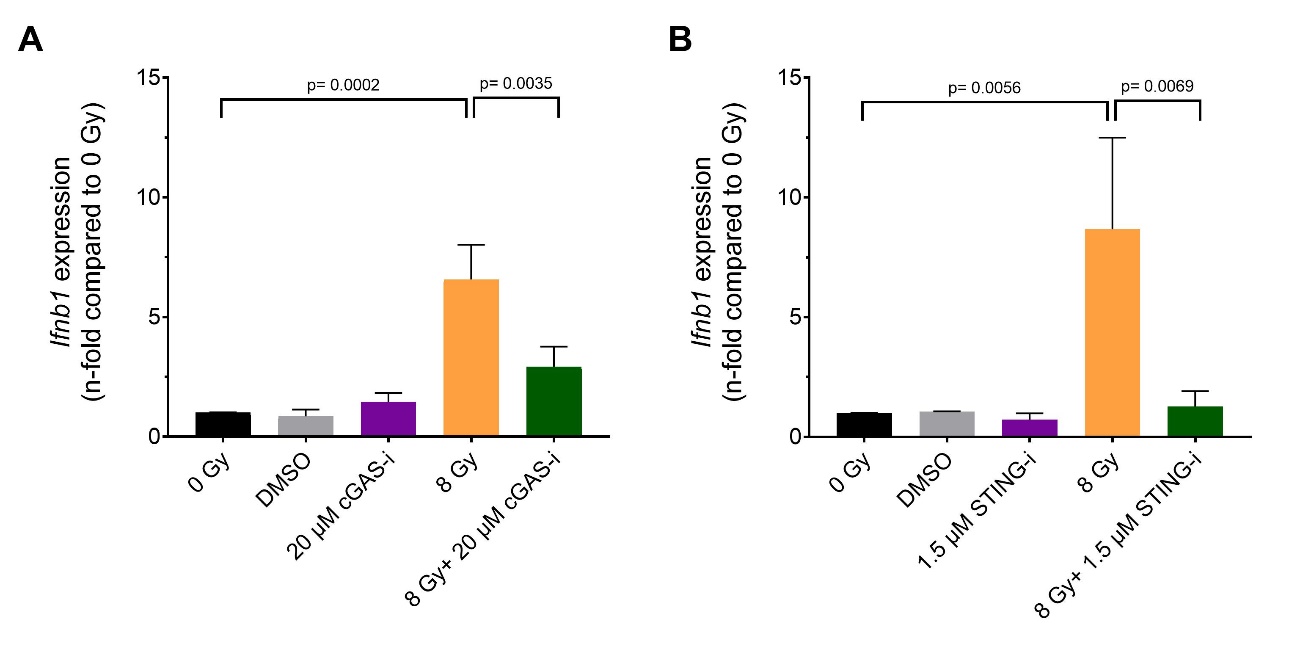


**Figure E7**. **Selective inhibition of cGAS or STING diminishes the expression of *Ifnb1* in 4T1 cells.** While exposure to 8 Gy X-rays results in an increased expression of *Ifnb1*, the effects are diminished applying selective inhibitors for cGAS (cGAS-i, A) or STING (STING-i, B) together with radiation exposure.


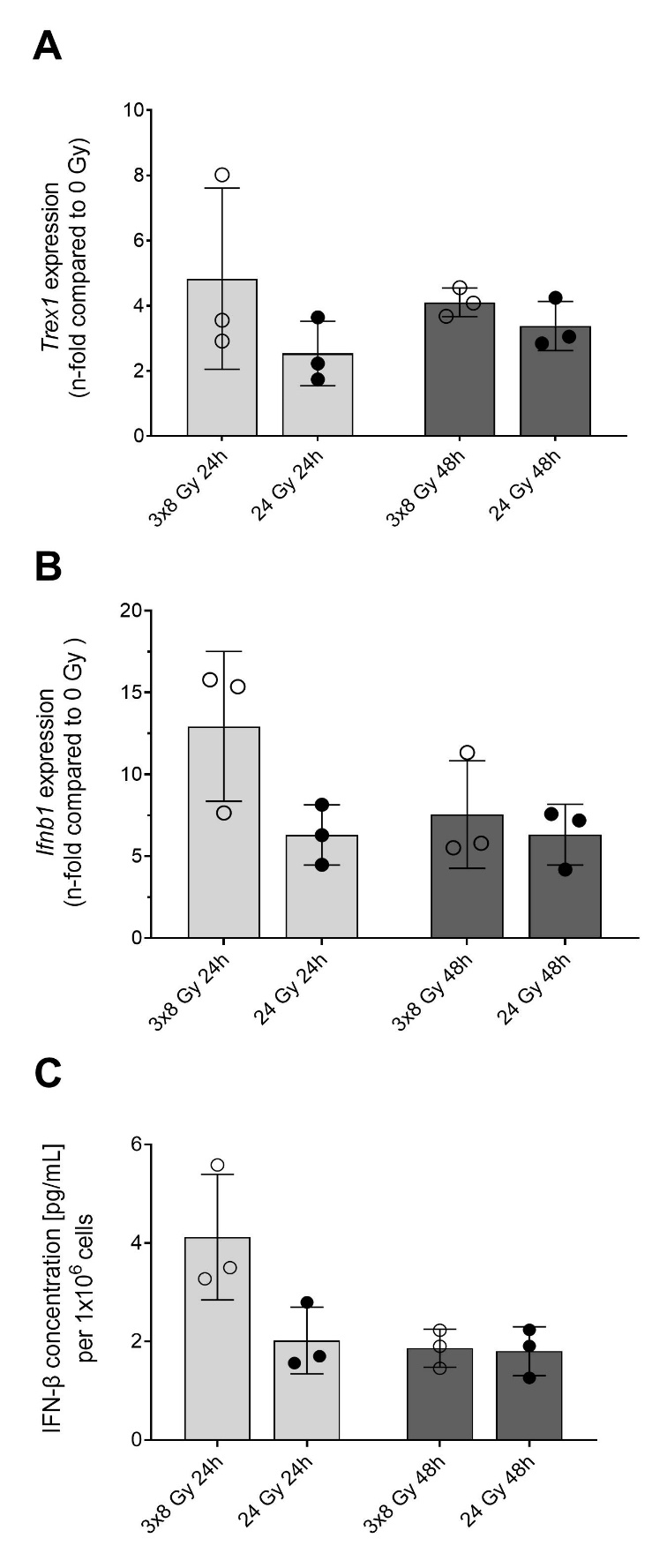


**Figure E8.** **Single high doses and a 3x8 Gy hypofractionation scheme result in similar levels of *Trex1* and *Ifnb1* expression and IFN-β release in TS/A cells.** The endpoints tested for the dose response curves were also performed comparing a single high dose of 24 Gy to a 3x8 Gy hypofractionation scheme at 24h and 48h after exposure to X-rays. More in detail, *Trex1* expression (A), *Ifnb1* expression (B), and IFN-β release (C) were measured. Significances were tested using an unpaired two-tailed t-test. None of the studied endpoints showed significant differences between single dose and fractionated dose.

**Supplementary Methods:**

Bulk RNA Sequencing

Total RNA from 4T1 and TS/A cells was quantified using a NanoDrop ND-1000 instrument. 1 to 2 µg of total RNA was used to prepare the sequencing library: the total RNA was enriched by oligo (dT) magnetic beads (rRNA removed); RNA-seq library was prepared using KAPA Stranded RNA-Seq Library Prep Kit (Roche), for a strand-specific RNA-seq library. The completed libraries were qualified with Agilent 2100 Bioanalyzer and quantified by absolute quantification qPCR method. To sequence the libraries on the Illumina NovaSeq 6000 instrument, the barcoded libraries were mixed, denatured to single stranded DNA in NaOH, captured on Illumina flow cell, amplified in situ, and subsequently sequenced for 150 cycles for both ends on Illumina NovaSeq 6000 instrument. Image analysis and base calling were performed using Solexa pipeline v1.8 (Off-Line Base Caller software, v1.8). Sequence quality was examined using the FastQC [1] software (version 0.11.7). The trimmed reads (trimmed 5’, 3’-adaptor bases using cutadapt [2] version 1.17) were aligned to reference genome using Hisat2 software [3] (version 2.1.0). The transcript abundances for each sample was estimated with StringTie [4] (version 1.3.3), and the FPKM alue for gene and transcript level were calculated with R package Ballgown [5–8] (version 2.10.0). The differentially expressed genes and transcripts were filtered using R package Ballgown. Fold change (cutoff 1.5), p-value (≤ 0.05) and FPKM (≥ 0.5 mean in one group) were used for filtering differentially expressed genes and transcripts. Principle Component Analysis (PCA) and correlation analysis were based on gene expression level, Hierarchical Clustering, Gene Ontology, Pathway analysis, scatter plots, and volcano plots were performed with the differentially expressed genes in R (version 3.5.0), Python (version 2.7) or shell environment for statistical computing and graphics. The Z-score hierarchical clustering heat maps were obtained for each Gene Of Interest (GOI) with the formula:

$Z-score= \frac{\left( FPKM sample-FPKM base mean \right)}{FPKM base SD}$

FPKM: fragments per kilobase of transcript per million mapped reads

FPKM sample: mean of FPKM values for the biological triplicates of the GOI

FPKM base mean: mean of FPKM values across all the samples for the GOI

FPKM base SD: standard deviation of FPKM values across all the samples for the GOI

**Table E3**: Quantification cycle (Cq) values of the reference genes used to calculate the fold change of the genes of interest. Data are taken from Fig. 2C and Supplementary Fig. 1A. The mean of three biological replicates and the standard deviation are reported.

| **Cell line** | **Radiation** | **Dose [Gy]** | ***Gapdh*** | ***Rpl13a*** |
| --- | --- | --- | --- | --- |
| 4T1 | X-rays | 0 | 16.008± 0.357 | 17.178± 0.432 |
|  |  | 2 | 16.273± 0.205 | 17.458± 0.167 |
|  |  | 4 | 16.244± 0.038 | 17.764± 0.043 |
|  |  | 8 | 16.401± 0.290 | 17.930± 0.131 |
|  |  | 20 | 16.335± 0.326 | 18.138± 0.309 |
|  | | | | |
|  | C-ions | 0 | 16.215± 0.182 | 18.511± 0.501 |
|  |  | 2 | 16.416± 0.122 | 18.691± 0.457 |
|  |  | 8 | 16.439± 0.405 | 18.610± 0.375 |
|  |  | 20 | 16.849± 0.183 | 19.290± 0.452 |
|  | | | | |
| TS/A | X-rays | 0 | 16.665± 1.077 | 18.580± 0.501 |
|  |  | 2 | 16.816± 1.028 | 19.058± 0.061 |
|  |  | 4 | 16.856± 0.700 | 19.369± 0.475 |
|  |  | 8 | 17.017± 1.542 | 19.621± 0.850 |
|  |  | 20 | 16.468± 0.439 | 19.208± 0.778 |

Best fit of the functions

Data in Figure 2A were fitted by the linear function:

$$Y=\alpha D$$

were Y is the number of cytoplasmic dsDNA foci/cell, α [Gy^-1^] is the slope and D the dose in Gy. The α values were 2.53±0.19 [Gy^-1^] for X-rays and 3.68±0.02 [Gy^-1^] for C-ions. The difference is statistically significant (t-test; p<0.0001).

We performed the fit of the experimental data in Figure 2 C-D for *Trex1* expression Y vs. dose D with the function:

$$Y=1+(P-1)(1-e^{-kD})$$

where k (Gy^-1^) is the slope of the curve, P the plateau value of the *Trex1* expression, D the dose in Gy.

The best fit values and their uncertainty are reported in Table S1:

**Table E2**: Fitting parameters for the data in Figure 2 C-D.

| **Time after exposure** | **Radiation** | **k±SD** | **P±SD** |
| --- | --- | --- | --- |
|  |  |  |  |
| **24 h** | X-rays | 0.10±0.07 | 3.1±1.0 |
|  | C-ions | 0.12±0.11 | 4.4±1.9 |
|  | p-value | 0.643 | 0.0662 |
| **48 h** | X-rays | 0.16±0.10 | 3.5±0.7 |
|  | C-ions | 0.34±0.21 | 5.1±0.6 |
|  | p-value | 0.0123 | <0.0001 |

An unpaired two-tailed t-test was used to test the difference of P and k parameters between X-rays and carbon ions´ fit. The p-values are shown in Table S1.

The same function was used to fit *Ifnb1* expression (Figure 4 A, C). However, there is no statistically significant difference between X-rays and C-ions. The fitting parameters are reported in Table S2:

**Table E3**: Fitting parameters for the data in Figure 4 A, C.

| **Time after exposure** | **Radiation** | **k** | **P** |
| --- | --- | --- | --- |
|  |  |  |  |
| **24 h** | X-rays | 0.13±0.07 | 8.65±1.75 |
|  | C-ions | 0.10±0.02 | 33.64±3.37 |
|  |  |  |  |
| **48 h** | X-rays | 0.02±0.02 | 254±217 |
|  | C-ions | 0.09±0.02 | 205±24 |
|  |  |  |  |

The IFN-β concentration data for X-rays and C-ions (Figure 4 B, D) seem to follow a different trend, with X-rays still increasing at 20 Gy while C-ions are in plateau. Therefore, we do not compare fitting parameters here.

Supplementary reference list

1. Andrews 2010, FastQC: A Quality Control Tool for High Throughput Sequence Data [Online], Available online at: <http://www.bioinformatics.babraham.ac.uk/projects/fastqc/>
2. Martin 2010, Cutadapt removes adapter sequences from high-throughput sequencing reads, Bioinformatics. 17(1), DOI: 10.14806/ej.17.1.200
3. Kim et al. 2015, HISAT: a fast spliced aligner with low memory requirements, DOI: 10.1038/nmeth.3317
4. Pertea et al. 2015, StringTie enables improved reconstruction of a transcriptome from RNA-seq reads, Nature Biotechnology. 33, p. 290-295, DOI: 10.1038/nbt.3122
5. Mortazavi et al. 2008, Mapping and quantifying mammalian transcriptomes by RNA-Seq. Nature Methods. 5(7):621-8, DOI: 10.1038/nmeth.1226
6. Fu et al. 2016, Ballgown: Flexible, isoform-level differential expression analysis. R package version 2.6.0.
7. Frazee et al. 2015, Ballgown bridges the gap between transcriptome assembly and expression analysis, Nature Biotechnology. 33(3): p. 243-246, DOI: 10.1038/nbt.3172
8. Pertea et al. 2008, Transcript-level expression analysis of RNAseq experiments with HISAT, StringTie and Ballgown. Nature Protocols. 11, p. 1650-1667, DOI: 10.1038/nprot.2016.095
